# Engineering a High-Fidelity MAD7 Variant with Enhanced Specificity for Precision Genome Editing via CcdB-Based Bacterial Screening

**DOI:** 10.3390/biom15101413

**Published:** 2025-10-04

**Authors:** Haonan Zhang, Ying Yang, Tianxiang Yang, Peiyao Cao, Cheng Yu, Liya Liang, Rongming Liu, Zhiying Chen

**Affiliations:** 1 Central Hospital of Dalian University of Technology, Xinan Road No. 826, Dalian 116024, China; zhanghaonan0715@mail.dlut.edu.cn (H.Z.); yaying1980128@163.com (Y.Y.); 2 MOE Key Laboratory of Bio-Intelligent Manufacturing, School of Bioengineering, Dalian University of Technology, Linggong Road No. 2, Dalian 116024, China; yangtx2024@mail.dlut.edu.cn (T.Y.); peiyaocao@mail.dlut.edu.cn (P.C.); yucheng@mail.dlut.edu.cn (C.Y.); liya_liang@dlut.edu.cn (L.L.)

**Keywords:** off-target, bacterial screening system, high specificity, MAD7_HF

## Abstract

CRISPR (clustered regularly interspaced short palindromic repeats)-Cas (CRISPR-associated protein) nucleases enable precise genome editing, but off-target cleavage remains a critical challenge. Here, we report the development of MAD7_HF, a high-fidelity variant of the MAD7 nuclease engineered through a bacterial screening system leveraging the DNA gyrase-targeting toxic gene *ccdB*. This system couples survival to efficient on-target cleavage and minimal off-target activity, mimicking the transient action required for high-precision editing. Through iterative selection and sequencing validation, we identified MAD7_HF, harboring three substitutions (R187C, S350T, K1019N) that enhanced discrimination between on- and off-target sites. In *Escherichia coli* assays, MAD7_HF exhibited a >20-fold reduction in off-target cleavage across multiple mismatch contexts while maintaining on-target efficiency comparable to wild-type MAD7. Structural modeling revealed that these mutations stabilize the guide RNA-DNA hybrid at on-target sites and weaken interactions with mismatched sequences. This work establishes a high-throughput bacterial screening strategy that allows the identification of Cas12a variants with improved specificity at a given target site, providing a useful framework for future efforts to develop precision genome-editing tools.

## 1. Introduction

The advent of CRISPR (clustered regularly interspaced short palindromic repeats)-Cas (CRISPR-associated protein) technology has revolutionized the field of genome editing, offering unprecedented precision and versatility in manipulating DNA sequences across all domains of life [1,2,3,4,5,6]. Among the diverse array of CRISPR-Cas nucleases identified to date, MAD7—a class 2 type V-A enzyme derived from the gut bacterium Eubacterium rectale—has emerged as a particularly promising tool. Distinguished by its compact size (~1200 amino acids), unique protospacer-adjacent motif (PAM) requirement (5′-YTTV), and broad activity across prokaryotic and eukaryotic systems, MAD7 has gained traction for applications ranging from basic genetic research to therapeutic development [7,8,9,10]. Its efficient mode of action, characterized by rapid target engagement and minimal lingering activity, further enhances its appeal, as this property minimizes prolonged nuclease exposure, reduces off-target effects, and avoids the risks associated with DNA-based delivery (e.g., insertional mutagenesis or immune responses) [11,12,13,14].

However, despite these advantages, MAD7, like all RNA-guided nucleases, is plagued by a critical limitation: off-target cleavage. Off-target effects arise when the MAD7-gRNA (guide RNA) complex tolerates mismatches between the gRNA and genomic DNA, leading to unintended double-stranded breaks (DSBs) at sites with partial sequence complementarity [15]. These errors are not merely technical nuisances; in therapeutic contexts, they can disrupt essential genes, trigger genomic instability, or even activate oncogenic pathways, undermining the safety and efficacy of editing strategies [16,17,18,19]. In research settings, off-target cleavage can confound experimental results, leading to misinterpretation of phenotypic changes and wasted resources [20,21,22].

Efforts to mitigate off-target activity have focused on multiple fronts [23,24,25]. gRNA design algorithms, for instance, prioritize sequences with minimal complementarity to non-target loci, but such approaches are constrained by the complexity of genomic landscapes and cannot eliminate cross-reactivity entirely. Chemical modifications of gRNAs—such as 2′-O-methylation [26] or phosphorothioate [27] linkages—can enhance stability and reduce off-target binding, but these modifications are costly and may vary in efficacy across cell types or target sites [28,29,30,31,32].

A more direct solution lies in engineering high-fidelity nuclease variants with intrinsic improvements in on-target specificity. For example, rational design of SpCas9 mutants (e.g., SpCas9-HF1 [33], eSpCas9(1.1) [34]) has yielded variants that reduce off-target cleavage by destabilizing interactions with mismatched DNA, while directed evolution approaches (e.g., HypaCas9 [35]) have selected for enzymes with enhanced proofreading capabilities. However, these variants often suffer from a trade-off: reduced off-target activity is accompanied by diminished on-target efficiency, particularly when delivered as ribonucleoprotein (RNP)—precisely the context where their specificity would be most valuable [36]. Compared with other delivery approaches, electroporation of RNPs introduces a large amount of pre-assembled RNPs into cells in a transient manner. Given the relatively short half-life of RNPs, this strategy results in significantly lower off-target effects under comparable conditions than plasmid- or mRNA-based gene editing methods.

To address this gap, we aimed to develop a screening strategy capable of rapidly isolating high-fidelity MAD7 variants that retain robust on-target activity while minimizing off-target cleavage, even under conditions mimicking transient nuclease action. Our approach leverages an unbiased bacterial screening system designed to recapitulate the rapid kinetics of nuclease activity, ensuring that selected mutants perform optimally in scenarios demanding high precision. Central to this system is a customizable toxin-based selection module that links cell survival to precise genome editing outcomes. The toxic gene is engineered such that its expression is lethal unless an on-target sequence embedded within the toxic plasmid is accurately cleaved. Concurrently, a co-maintained plasmid encodes chloramphenicol resistance, but only if an off-target sequence (differing from the on-target by a single nucleotide) remains intact. This dual-selection mechanism ensures that survivors must both cleave the on-target site to prevent toxin expression and avoid cleaving the off-target site to retain antibiotic resistance. Thus, only nucleases capable of efficient and specific editing can relieve the cytotoxic pressure and permit survival. This design allows flexible adaptation to different toxic backbones and was optimized to enforce stringent selection under transient nuclease activity.

To further evaluate the feasibility of this system, we selected a locus within the human *TTR* gene as the target site and cloned it into a plasmid harboring a toxic gene. In parallel, we designed an artificial off-target site differing by only a single nucleotide and cloned it into a plasmid for protein expression. Using this dual-plasmid system, we screened a large MAD7 mutant library (~250,000 clones) generated via error-prone PCR, with the goal of identifying substitutions that enhance on-target and off-target discrimination. Candidates were subjected to rigorous validation, including deep sequencing of on-target loci to assess cleavage efficiency and targeted analysis of off-target sites with varying mismatch patterns. Structural modeling of lead variants aimed to unravel the mechanistic basis of their enhanced specificity, focusing on how mutations influence interactions with the gRNA, target DNA, and mismatched off-target sequences. By prioritizing performance under transient activity conditions and validating activity across diverse mismatch contexts, we aim to provide a tool that advances both basic research and therapeutic genome editing, where precision and safety are paramount.

## 2. Materials and Methods

### 2.1. Construction of the Dual-Plasmid Bacterial Screening System

The Cas12a nuclease ErCas12a (MAD7) along with the toxic gene used in this study were obtained from NCBI and synthesized by GENEWIZ (Azenta Life Sciences, Suzhou, China) with codon optimization for *Eschericha coli* (*E. coli*) usage. We cloned MAD7 under the control of the PL promoter into the pSC101 plasmid and introduced the designed off-target site via site-directed mutagenesis, generating the construct referred to as the expression plasmid. Concurrently, we used the NEBuilder^®^ HiFi DNA Assembly Master Mix (New England Biolabs, MA, USA) to insert a toxin gene under the control of an inducible promoter into the plasmid pBBR1MCS-2 (Addgene plasmid #85168). An on-target site was introduced via site-directed mutagenesis, and the resulting construct was designated as the toxic plasmid.

### 2.2. Design of On-Target and Off-Target Site

Cas-Designer(BAE Lab, Seoul National University College of Medicine, Seoul, South Korea; online version accessed on 14 September 2024) [37] was used to identify potential YTTV PAM-targetable sites within the *TTR* gene. To maximize patient applicability, target sites were selected outside the regions harboring known *TTR* gene mutations, based on data compiled from NCBI. Off-target analysis was performed using Cas-OFFinder(BAE Lab, Seoul National University College of Medicine, Seoul, South Korea; online version accessed on 14 September 2024) [38] to ensure that no additional matching sites existed elsewhere in the genome.

### 2.3. Preparation of Electrocompetent E. coli Cells and Electroporation

*E. coli* cells were first grown overnight and subsequently diluted 1:100 into fresh LB medium. Cultures were incubated at 37 °C with shaking at 200 rpm until reaching an OD_600_ of 0.55–0.6. Cells were then placed on ice for 15 min, followed by two rounds of centrifugation to remove the supernatant. The cell pellet was resuspended in 10% glycerol at 50% of the original culture volume and finally adjusted to a final volume equivalent to 1% of the initial culture volume to obtain electrocompetent cells.

For non-library electroporation experiments, 50 µL of electrocompetent cells were mixed with 100 ng of plasmid DNA in a pre-chilled 0.1 cm-gap electroporation cuvette (on ice for at least 15 min). Electroporation was performed at 1800 V, followed immediately by the addition of SOB medium. Cells were recovered at 30 °C with shaking at 200 rpm for 2 h, then incubated at 42 °C and 180 rpm for an additional 15 min.

### 2.4. Construction of MAD7 Mutation Library

MAD7 was mutagenized using the GeneMorph II Random Mutagenesis Kit (Agilent Technologies, CA, USA) with a targeted mutation frequency of 5–10 mutations per kilobase. A total of 0.15 pmol of backbone and a fivefold molar excess of the mutagenized fragment were assembled using the NEBuilder^®^ HiFi DNA Assembly Master Mix (New England Biolabs, Inc., Ipswich, MA, USA) at 50 °C for 4 h. The reaction product was desalted by floating a 0.025 µm pore filter on ddH_2_O. Electrocompetent cells were prepared as described above. For electroporation, 200 µL of cells were mixed with the assembly product in a 0.2 cm-gap cuvette and subjected to electroporation at 2500 V. Cells were immediately recovered in SOB medium and incubated at 30 °C, 200 rpm for 2 h. Subsequently, 10 µL of the recovery culture was plated on LB agar supplemented with 34 μg/mL chloramphenicol for overnight incubation. The remaining culture was expanded overnight, and plasmids were extracted the following day.

### 2.5. Procedure of Dual-Plasmid Bacterial Screening System

We first optimized the dual-plasmid selection system by electroporating *E. coli* electrocompetent cells with either the SacB-based or CcdB-based toxic plasmid. After electroporation, cells were incubated at 37 °C with shaking at 200 rpm for 1 h. The recovery cultures were then plated on selective LB agar containing 50 μg/mL kanamycin supplemented with varying concentrations of sucrose (for the SacB system) or 40 µg/L anhydrotetracycline (aTc) (for the CcdB system), as well as on control LB agar containing only 50 μg/mL kanamycin and colony-forming units (CFU) were subsequently counted. The killing efficiency was calculated using the following equation:

(1) $$\mathrm{Killing}\;\mathrm{efficiency}\left(\%\right)=\frac{{\mathrm{CFU}}_{\mathrm{selective}}}{{\mathrm{CFU}}_{\mathrm{control}}}\times 100\%$$

The toxic plasmid was first electroporated into electrocompetent *E. coli* cells and plated on LB agar supplemented with 50 μg/mL kanamycin. Electrocompetent cells were then prepared from *E. coli* harboring the toxic plasmid, and the expression plasmid was electroporated into these cells. After electroporation, cells were incubated at 30 °C and 200 rpm for 2 h, followed by an additional 15 min incubation at 42 °C and 180 rpm to induce expression of the MAD7 protein. The cultures were then plated on LB agar containing 34 μg/mL chloramphenicol and the appropriate inducer for overnight incubation. Colonies that appeared on the plates were considered to have passed the selection. These colonies were scraped from the plates for plasmid extraction and reintroduced into *E. coli* electrocompetent cells containing the toxic plasmid. The selection process was then repeated for a second round.

### 2.6. Off-Target Cleavage and System Survival Assays for MAD7 Variants

For the off-target assay, plasmids encoding all selected MAD7 variants were extracted. Expression plasmids carrying WT-MAD7 (wild type-MAD7) and WT-MAD7 lacking the off-target site were included as controls. For each electroporation, 100 ng of plasmid was introduced into *E. coli* electrocompetent cells. After electroporation, cells were incubated at 30 °C and 200 rpm for 2 h, followed by incubation at 42 °C and 180 rpm for an additional 15 min to induce MAD7 expression. Cultures were plated on LB agar containing 34 μg/mL chloramphenicol and 40 µg/L anhydrotetracycline for overnight incubation, and CFU were counted. The off-target cleavage efficiency was calculated using the following equation:

(2) $$\mathrm{Off}\text{-}\mathrm{target}\;\mathrm{efficiency}\left(\%\right)=\frac{{\mathrm{CFU}}_{\mathrm{without}\;\mathrm{off}\text{-}\mathrm{target}}\;-{\mathrm{CFU}}_{\mathrm{variant}}}{{\mathrm{CFU}}_{\mathrm{without}\;\mathrm{off}\text{-}\mathrm{target}}}\;\times \;100\%$$

For the on-target cleavage assays, expression plasmid carrying WT-MAD7 was used as the control. Electrocompetent *E. coli* cells harboring the toxic plasmid were prepared as previously described. Plasmids encoding different MAD7 variants were electroporated into these cells. Following electroporation, cells were recovered at 30 °C and 200 rpm for 2 h, followed by incubation at 42 °C and 180 rpm for an additional 15 min to induce MAD7 expression. Cultures were plated on LB agar containing 34 μg/mL chloramphenicol and LB agar containing 34 μg/mL chloramphenicol and 50 μg/mL kanamycin, followed by overnight incubation. CFUs were then counted. On-target efficiency was determined based on colony counts on selective and non-selective plates, as defined below:

(3) $$\mathrm{On}\text{-}\mathrm{target}\;\mathrm{efficiency}\left(\%\right)=\frac{{\mathrm{CFU}}_{\mathrm{CHL}}\;-{\mathrm{CFU}}_{\mathrm{CHL}+\mathrm{Kan}}}{{\mathrm{CFU}}_{\mathrm{CHL}}}\times 100\%$$

To compare the activity of MAD7 variants with the WT-MAD7, relative on-target activity was calculated as follows:

(4) $$\mathrm{Relative}\;\mathrm{on}\text{-}\mathrm{target}\;\mathrm{activity}\;(\mathrm{vs}.\;\mathrm{WT}\text{-}\mathrm{MAD}7)\;=\;\frac{{\mathrm{On}\text{-}\mathrm{target}\;\mathrm{efficiency}}_{\mathrm{Variant}}}{{\mathrm{On}\text{-}\mathrm{target}\;\mathrm{efficiency}}_{WT\text{-}\mathrm{MAD}7}}$$

### 2.7. Structure Prediction of MAD7_HF

To compare structural features, ternary complexes were predicted using AlphaFold3 (DeepMind, London, UK; online version accessed on 31 May 2025) for both WT-MAD7 and MAD7_HF, each assembled with the same gRNA and target DNA [39]. We used the AlphaFold server, inputting the sequences of the protein, gRNA, and DNA, and generated the model directly with AlphaFold3 under default parameters without any optimization. For mutation sites located within 6 Å of the gRNA or dsDNA, hydrogen bonds were annotated to visualize interaction networks before and after mutation, highlighting potential changes in the molecular interface between MAD7 and its nucleic acid substrates.

## 3. Results

### 3.1. Development of a Dual-Plasmid Bacterial Screening System for High-Fidelity MAD7 Mutants Based on Toxin Gene

**Figure 1:**
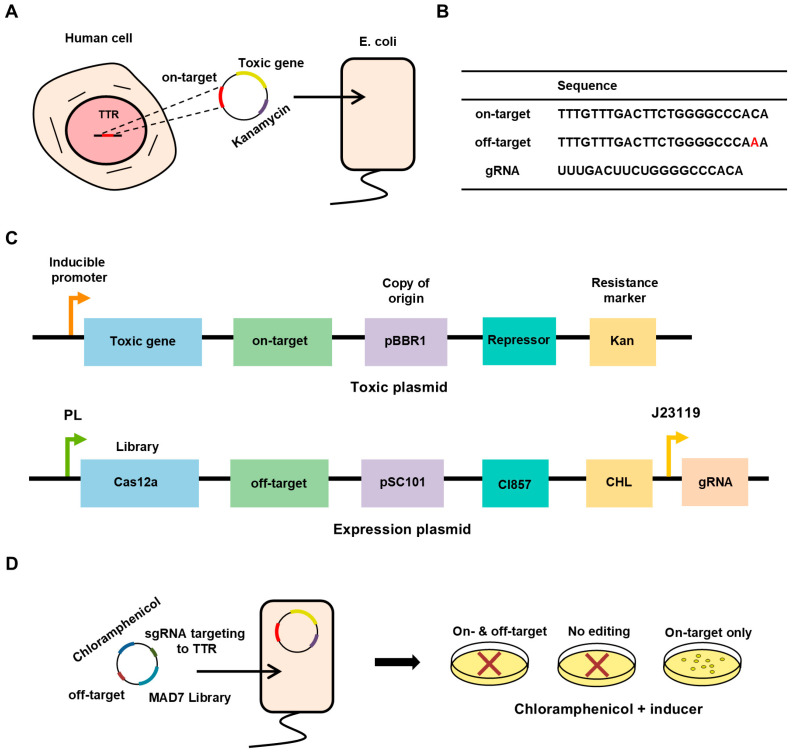
Workflow and principle of the dual-plasmid selection system in *E. coli*. (**A**) Selection of the target sequences from the human genome. (**B**) Design of on-target, off-target, and gRNA sequences. (**C**) Schematic diagram of the dual-plasmid system. (**D**) Selection principle based on editing specificity.

To identify MAD7 variants with enhanced specificity while preserving on-target activity, we established a dual-plasmid bacterial screening system centered on a toxic gene (Figure 1A). This system relies on two plasmids with distinct origins of replication to ensure stable co-maintenance in *E. coli*: the “expression plasmid” (pSC101 origin) and the “toxic plasmid” (pBBR1 origin). For this study, we selected the human TTR locus as a model target, as it is clinically relevant to genome editing research. The *TTR* gene encodes a plasma transport protein, and its mutations are closely associated with hereditary ATTR (transthyretin amyloidosis). ATTR is a progressive, often fatal rare disease caused by the deposition of misfolded TTR proteins as amyloid fibrils in tissues such as the heart and nerves, making the *TTR* gene a valuable target for therapeutic genome editing.

The expression plasmid harbors a MAD7 mutant library, a guide RNA (gRNA) targeting the human TTR locus (sequences: UUUGACUUCUGGGGCCCACA), and a chloramphenicol resistance marker. Expression of MAD7 is regulated by the CI857 repressor under the PL promoter, enabling tight control over their production and the gRNA is expressed from the strong constitutive promoter J23119, ensuring sufficient guide RNA levels to support efficient cleavage. For all strains carrying the expression plasmid, unless a specific incubation temperature was indicated (e.g., 42 °C induction), the cultures were maintained at 30 °C to ensure that cI857 effectively repressed Cas12a expression. An off-target site (TTTGTTTGACTTCTGGGGCCCAAA) differing by a single nucleotide at position 19 from the 5′ end (second-to-last base), corresponding to a distal mismatch. The toxic plasmid carries a toxic gene, along with an on-target site (TTTGTTTGACTTCTGGGGCCCACA) matching *TTR* (Figure 1B). Expression of the toxic gene is likewise controlled by a tightly regulated, inducible promoter (Figure 1C).

**Figure 2:**
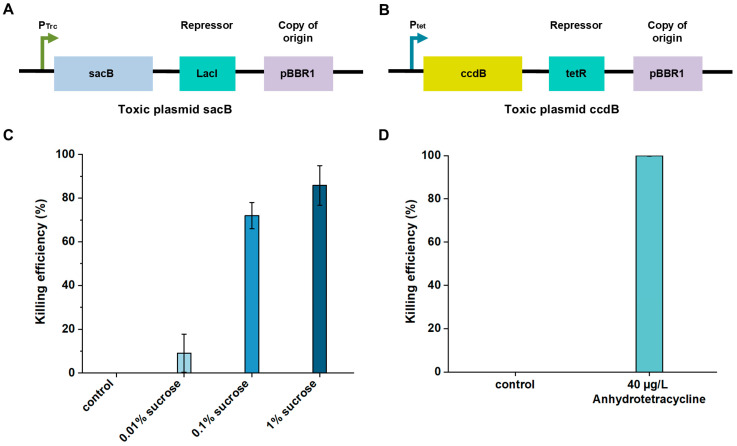
Killing efficiency of toxic plasmids based on different toxic genes. (**A**) Schematic of the toxic plasmid utilizing *sacB* as the toxic gene. (**B**) Schematic of the toxic plasmid utilizing *ccdB* as the toxic gene. (**C**) Killing efficiency of SacB-based toxic plasmid under different sucrose concentrations. (**D**) Killing efficiency of CcdB-based toxic plasmid under anhydrotetracycline induction. n = 3 for each experiment. Error bars represent mean values ± SD.

For a mutant to survive selection, it must fulfill two criteria: (i) cleave the on-target site to inactivate the toxic gene (enabling survival in inducer) and (ii) avoid cleaving the off-target site (to retain chloramphenicol resistance) (Figure 1D). To further enhance the specificity of the screening system, we implemented two distinct toxin-based selection strategies (SacB and CcdB). The first strategy utilizes the sucrose-sensitive *sacB* gene under the control of a Trc promoter (Figure 2A); the *sacB* gene encodes an enzyme that converts sucrose into fructan polymers that are lethal to *E. coli*. The second strategy employs the *ccdB* gene driven by a tetracycline-inducible promoter (Figure 2B); the *ccdB* gene encodes a toxin protein that targets DNA gyrase by stabilizing the gyrase–DNA cleavage complex, thereby interfering with DNA replication. We validated the cytotoxicity of both selection systems to ensure that only cells in which the toxic plasmid was successfully cleaved could survive, thereby enabling stringent elimination of MAD7 variants with reduced or lost activity. In the SacB-based system, the optimized sucrose concentration led to a killing efficiency of 85.5%, reflecting effective SacB-mediated cytotoxicity (Figure 2C), whereas the CcdB-based system achieved a markedly higher killing efficiency of 99.9% (Figure 2D).

### 3.2. Isolation of MAD7_HF, a High-Fidelity MAD7 Mutant with Reduced Off-Target Activity via ccdB Selection

**Figure 3:**
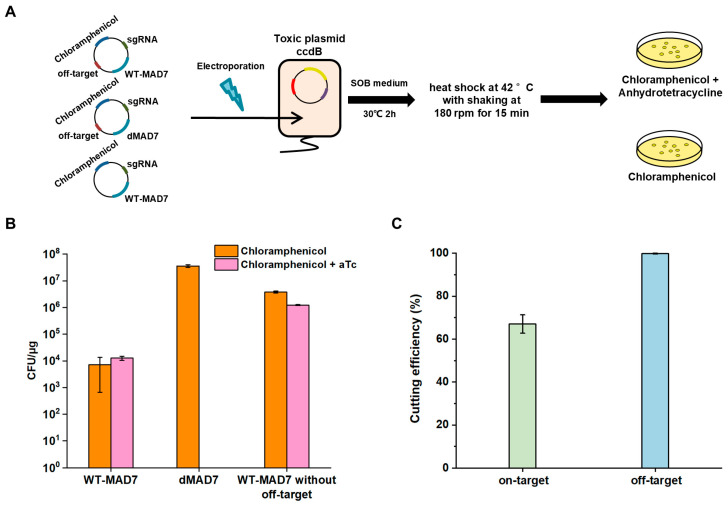
Screening performance of the dual-plasmid system based on the CcdB toxic plasmid. (**A**) Workflow of the CcdB-based dual-plasmid screening system using different expression plasmids. (**B**) Screening outcome using different expression plasmids under induced and non-induced conditions. (**C**) Cutting efficiency of WT-MAD7 at on-target and off-target sites. *n* = 3 for each experiment. Error bars represent mean values ± SD.

To assess the efficiency of the screening system, we introduced expression plasmids encoding wild-type MAD7 (WT-MAD7), dead MAD7 (dMAD7), and WT-MAD7 lacking the off-target site into the dual-plasmid selection platform. These constructs were plated on agar containing chloramphenicol alone or chloramphenicol supplemented with aTc (Figure 3A). The results demonstrated that the screening system effectively eliminated MAD7 variants with lost nuclease activity (Figure 3B). WT-MAD7 achieved a 67% cleavage efficiency on the toxic plasmid, thereby inactivating the toxic gene; however, its pronounced off-target activity led to a 99.8% cleavage rate on the expression plasmid (Figure 3C). This high off-target activity resulted in a markedly low survival rate of WT-MAD7 on the selective plates, highlighting the stringent nature of the system. These findings provide a strong rationale for employing the dual-plasmid system to enrich for MAD7 variants with both high fidelity and robust on-target activity.

We screened a MAD7 mutant library (~250,000 clones) using the CcdB-based dual-plasmid system, with selection pressure from chloramphenicol and aTc. After two rounds of selection, 57 candidate mutants were identified. During the expansion of selected mutants, we observed that some colonies exhibited poor growth on plates containing chloramphenicol. We hypothesized that leaky expression of certain MAD7 variants during colony growth may have led to cleavage at the off-target site present on the expression plasmid, due to interaction with the co-expressed gRNA. As a result, strains harboring MAD7 variants with high off-target activity were unable to maintain the expression plasmid and thus failed to grow under antibiotic selection. Ultimately, we successfully isolated 38 candidate mutants for subsequent evaluation of on-target efficiency and off-target specificity.

### 3.3. Validation of On-Target and Off-Target Activity of MAD7 in Bacterial Assays

**Figure 4:**
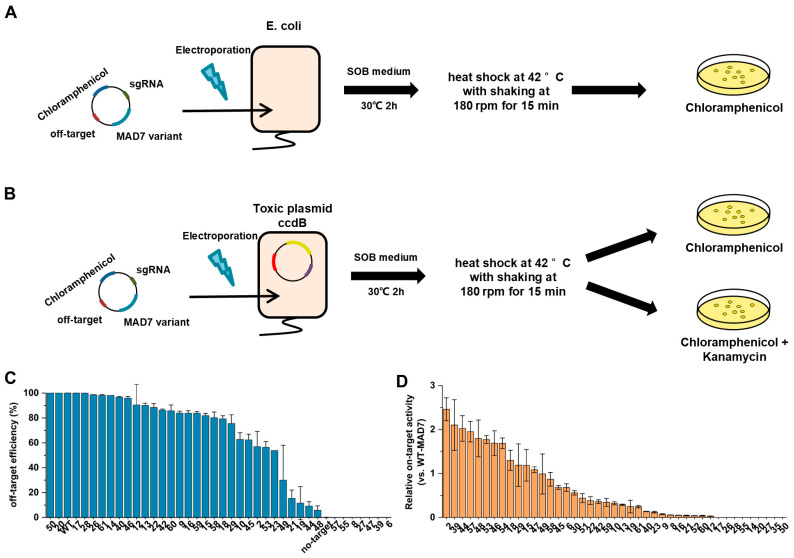
Evaluation of On-target and Off-target Activities of MAD7 Variants. (**A**) Workflow for off-target activity assessment of MAD7 variants. (**B**) Workflow for on-target activity assessment of MAD7 variants. (**C**) Off-target editing efficiencies of various MAD7 variants (**D**) Relative on-target activity of MAD7 variants compared to WT-MAD7. *n* = 3 for each experiment. Error bars represent mean values ± SD.

To rigorously confirm the specificity and efficiency of MAD7 mutants, we performed detailed on-target and off-target validation using *E. coli* as the host system (Figure 4A,B). For off-target validation, we focused on the pre-identified off-target site (TTTGTTTGACTTCTGGGGCCCAAA), which differs from the on-target sequences by a single nucleotide. Expression plasmids encoding different MAD7 mutants and corresponding off-target sequence were electroporated into *E*. *coli*. Following recovery, cells were plated on chloramphenicol-containing LB agar, and transformation with expression plasmid lacking the off-target sequence served as a control (Figure 4A and Supplementary Figure S1). The cleavage efficiency at off-target sites was quantified for each MAD7 variant. Compared to WT-MAD7, most mutants exhibited reduced editing activity. Notably, 17 variants showed a greater than 30% decrease in off-target cleavage efficiency, and for 7 mutants, off-target activity was undetectable under these assay conditions.

For on-target validation, we employed a plasmid-based system containing an on-target site and a kanamycin resistance marker to assess cleavage efficiency. Expression plasmids encoding different MAD7 mutants were electroporated into *E. coli* strains harboring this plasmid. Following electroporation, cells were incubated at 42 °C for 15 min to induce MAD7 expression. Cells were then plated on two types of agar: one supplemented with chloramphenicol alone and the other with both chloramphenicol and kanamycin. Survival on these media directly correlates with the integrity of the reporter plasmid. If the on-target site is cleaved, the resistance gene is disrupted resulting in cell death on kanamycin-containing agar. A wild-type MAD7 construct served as a reference control (Figure 4B and Supplementary Figure S2). Among the tested variants, 18 MAD7 mutants exhibited higher on-target efficiency than WT-MAD7 with 9 of them achieving complete 100% cleavage of the on-target site. Notably, MAD_39 demonstrated a 2.1-fold increase in on-target efficiency compared to WT-MAD7 while its off-target activity remained undetectable under these conditions. This variant was subsequently designated as MAD7_HF.

### 3.4. Structural Basis for the High Fidelity of MAD7_HF

**Figure 5:**
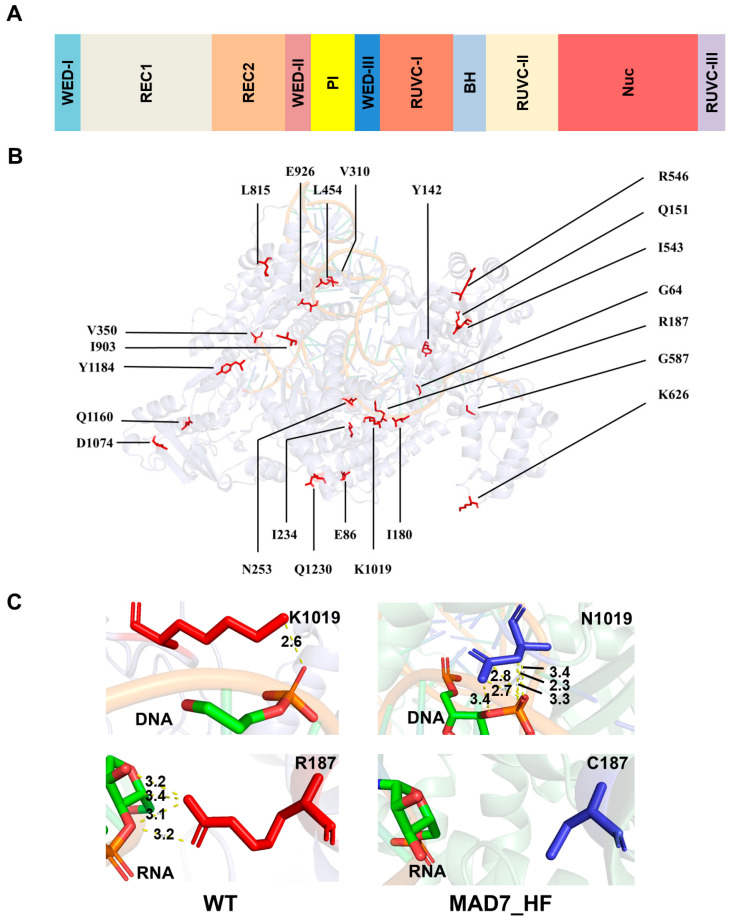
Structural Analysis of MAD7_HF. (**A**) Domain organization of MAD7. (**B**) Ternary complex structure of MAD7–gRNA–DNA and spatial distribution of all beneficial mutations on WT-MAD7. (**C**) Interactions between MAD7-HF mutation sites and DNA/RNA before and after mutation.

11 MAD7 variants exhibiting higher editing efficiency and specificity than WT-MAD7 were subjected to Sanger sequencing (Table 1). All gain-of-function mutants were sequenced and analyzed based on the domain architecture of MAD7 (Figure 5A). The identified mutations were subsequently mapped onto a structure of MAD7 generated by AlphaFold3. Structural analysis revealed that these mutations are distributed across key functional domains of the protein (Figure 5B).

The MAD7_HF, which exhibited the highest editing efficiency and specificity, harbored three mutations: R187C, S350T, and K1019N. After modeling the MAD7_HF–DNA–RNA ternary complex using AlphaFold3, we analyzed the spatial distances between the mutation sites and the DNA/RNA to infer their potential interaction relationships (Figure 5C). R187 is located in the REC domain, which mediates interactions with the RNA-DNA hybrid. The substitution of arginine (positively charged) with cysteine (neutral) weakens non-specific electrostatic interactions with the phosphate backbone of off-target DNA (which contains mismatches), reducing off-target binding. K1019, in the nuclease domain, forms a hydrogen bond with the gRNA backbone; its mutation to asparagine (K1019N) stabilizes this interaction, ensuring the gRNA remains properly aligned only when fully complementary to the target. Substitution of serine with threonine at position 350 in the WED domain introduces an additional methyl group, which may cause steric hindrance or alter the local hydrogen-bonding network. This mutation could delay or attenuate the conformational transition of Cas12a from the recognition to the activation state, thereby increasing the activation energy threshold and enhancing the specificity of MAD7. Together, these changes enable MAD7_HF to distinguish between on- and off-target sites while preserving on-target activity.

## 4. Discussion

The *E. coli* high-throughput screening system we established, based on the CcdB toxin, provides a versatile strategy for the directed evolution of CRISPR genome-editing enzymes. By enabling the rapid selection of variants with reduced off-target effects, this platform directly addresses one of the major challenges limiting both research and therapeutic applications. In this study we applied an unbiased, CcdB-based bacterial selection to isolate MAD7_HF, a MAD7 variant that was identified and characterized specifically at a single locus within the human *TTR* gene, indicating a strong site-specific effect. Therefore, nuclease variants obtained through this screening strategy may be restricted in their applicability to the specific target sites used during selection. Nevertheless, this characteristic also highlights the strength of the system, namely its ability to generate “precision medicine-like” nucleases tailored for individual loci, thereby offering new opportunities for highly accurate gene editing at specific targets.

The dual-plasmid bacterial screening system we developed offers a powerful alternative to rational design or in vitro selection methods for engineering nuclease specificity. By coupling survival to two orthogonal criteria—efficient on-target cleavage (to inactivate *ccdB* and resist toxicity) and minimal off-target cleavage (to retain chloramphenicol resistance)—the system directly enriches for mutants with enhanced on-target and off-target discrimination. This design mimics the transient activity of RNP delivery, where nuclease exposure is short-lived, ensuring that selected variants perform optimally in clinically relevant contexts. Compared to plasmid-based expression systems, which sustain nuclease production over time, our CcdB-based screen selects for mutants that act quickly and specifically. This is critical because prolonged nuclease activity can mask deficits in on-target efficiency, as seen with some high-fidelity Cas9 variants that perform well in plasmid-based assays but fail in RNP delivery. By contrast, MAD7_HF’s strong performance in our screen—with 85% survival under high-stringency conditions—directly translates to robust activity in transient contexts, making it suitable for ex vivo and in vivo editing where RNPs are the delivery method of choice. Furthermore, the scalability of the system allows for screening large mutant libraries (~250,000 clones), increasing the likelihood of identifying rare variants with subtle but impactful mutations. This unbiased approach bypasses limitations of rational design, which relies on prior structural or mechanistic insights that may not fully capture the dynamic interplay between nuclease, guide RNA, and target DNA.

The three substitutions in MAD7_HF—R187C, S350T, and K1019N—collectively enhance specificity through complementary mechanisms. These conclusions were derived from structural insights obtained by predicting the Cas12a–RNA–DNA ternary complex using AlphaFold3, which enabled us to infer the potential roles of these substitutions. R187, located in the REC domain, plays a key role in stabilizing the RNA-DNA hybrid during target recognition. The substitution of arginine (positively charged) with cysteine (neutral) reduces electrostatic interactions with the phosphate backbone of off-target DNA, which contains mismatches that disrupt the regular structure of the hybrid. This weakens non-specific binding without compromising on-target engagement, where base pairing is complete and the hybrid structure is stable. K1019, in the nuclease domain, forms a hydrogen bond with the guide RNA backbone; mutation to asparagine strengthens this interaction, ensuring the guide RNA remains properly aligned only when fully complementary to the target. This increases the energetic cost of tolerating mismatches, further reducing off-target cleavage. S350T, in the PI domain, may enhance guide RNA loading or stability, indirectly supporting precise target recognition by ensuring the nuclease-guide complex adopts an active conformation only at on-target sites. Together, these mutations strike a balance: they reduce promiscuous binding to off-target sites while preserving the interactions required for efficient on-target cleavage, addressing the longstanding trade-off between specificity and activity in high-fidelity nucleases.

The identification of MAD7_HF through our bacterial screening strategy demonstrates the utility of this method for discovering high-fidelity Cas12a variants. MAD7_HF’s performance in bacterial assays underscores its potential for diverse genome editing applications. Its ability to reduce off-target cleavage by >20-fold across multiple mismatch contexts—while maintaining on-target efficiency comparable to wild-type MAD7—makes it particularly valuable for RNP-based editing, where transient nuclease activity demands maximal on-target efficiency within a narrow timeframe. While our evaluation was limited to bacterial assays, the ability of MAD7_HF to reduce off-target cleavage while retaining robust on-target activity highlights its potential as a candidate for further investigation. Importantly, this work establishes a methodological framework that may facilitate the development of high-precision nucleases for future genome editing applications, including therapeutic settings where safety and specificity are critical.

Despite these strengths, our study has limitations that warrant further investigation. First, the CcdB-based selection system was applied only to a single target site and an artificially designed off-target site within the *TTR* gene. As a result, the variants obtained in this study are expected to exhibit high specificity primarily at this particular locus. While further validation at additional target sites would be required to fully demonstrate the general high specificity of MAD7_HF, this site-specific optimization approach highlights the potential of our method and provides a framework that could, in principle, be adapted to other loci in future studies. Second, while bacterial assays provide a robust initial validation, testing MAD7_HF in eukaryotic cells, particularly primary cells and animal models, is essential to confirm its translational potential. Eukaryotic genomes are more complex, and chromatin structure or epigenetic modifications may influence MAD7_HF’s activity differently than in bacterial systems. Moreover, the screening strategy established here is not limited to *E. coli*; it can be readily adapted to other prokaryotic hosts and, with appropriate optimization, extended to eukaryotic systems, which may further lower the barrier to translating screened variants into practical applications. Third, some MAD7 variants have already been applied in genome editing. Notably, the mutation sites of MAD7_HF differ from those of previously reported MAD7 variants, suggesting that combining these substitutions with high-fidelity mutations identified in other Cas12a nucleases may further enhance the specificity of MAD7. Then, combining MAD7_HF with other specificity-enhancing strategies—such as chemically modified guide RNAs or truncated guides—could further reduce off-target effects, and these synergies merit exploration. Finally, high-resolution structural studies of MAD7_HF in complex with on- and off-target DNA would provide atomic-level insights into how the identified mutations alter protein-DNA interactions, guiding rational design of next-generation nucleases.

## 5. Conclusions

In conclusion, this study highlights the value of a CcdB-based high-throughput screening strategy in *E. coli* for rapidly identifying Cas12a variants with improved specificity. The successful isolation of MAD7_HF demonstrates the effectiveness of this approach and underscores its potential as a general framework for nuclease optimization.

## Figures and Tables

**Table 1:** Distribution of mutation sites in MAD7 advantage mutants.

**Mutant MAD7 ID**	**Mutant Sites**
2	E86D
39	R187C, S350T, K1019N
4	Y142H, Q152H
57	D1074G, Q1230H
48	I234M, L815V, I903F
54	I543V, R546H
46	G96R
54	K626R, E926R
18	Q1160K, Y1184C
29	I180M
15	N253I, V310L, L454I, G587S

## Data Availability

The original contributions presented in this study are included in the article/Supplementary Materials. Further inquiries can be directed to the corresponding author.

## Supplementary Materials

The following supporting information can be downloaded at: https://www.mdpi.com/article/10.3390/biom15101413/s1, Table S1: Primer sequences used in this study; Figure S1: Representative experimental validations of off-target effects; Figure S2: Representative experimental validations of on-target effects.

## Abbreviations

The following abbreviations are used in this manuscript:

PAM	Protospacer-adjacent motif
CRISPR	Clustered regularly interspaced short palindromic repeats
Cas	CRISPR-associated protein
gRNA	Guide RNA
DSBs	Double-stranded breaks
RNP	Ribonucleoprotein
CFU	Colony-forming units
aTc	anhydrotetracycline
