# Supplementary material for: Engineering a High-Fidelity MAD7 Variant with Enhanced Specificity for Precision Genome Editing via CcdB-Based Bacterial Screening
Source: Biomolecules. 2025 Oct 4;15(10):1413. doi: 10.3390/biom15101413 (PMC12563919; doi:10.3390/biom15101413)
Supplement: Supplementary file 1 [file biomolecules-15-01413-s001.zip › biomolecules-3840770-supplementary.pdf]

Supplementary Materials

Table S1. Primer sequences used in this study.

Name	Sequence
SDM-toxic -on-target-F	TTGTTTGACTTCTGGGGCCCACAgaccgtgtgcttctcaaatgc
SDM-toxic -on-target-R	GCCCCAGAAGTCAAACAAAacactgcttccggtagtcaataaa
SDM-Expression-off-target-F	ttgtttgacttctggggcccaaATGCTCAAATTGGAATCAGGTTTG
SDM-Expression-off-target-R	tgggccccagaagtcaaaCAAAGAAGCACAGTGCTACGTGT
Error-MAD7-F	gcaacagttcatcgtcaagaacggaataat
Error-MAD7-R	gagatagcgcttattctggataaagtcgaacc
bb-Expression-F	tccagaataagcgctatctctaag
bb-Expression-R	cttgacgatgaactgttgcg
ID-MAD7-F1	acgcactgaccaccatgaag
ID-MAD7-F2	ttacccaggaaggcattagc
ID-MAD7-F3	gacgttagcagacggttgg
ID-MAD7-R1	cctgattccaatttgagca
ID-MAD7-R2	gatattcacaaagccggtgg

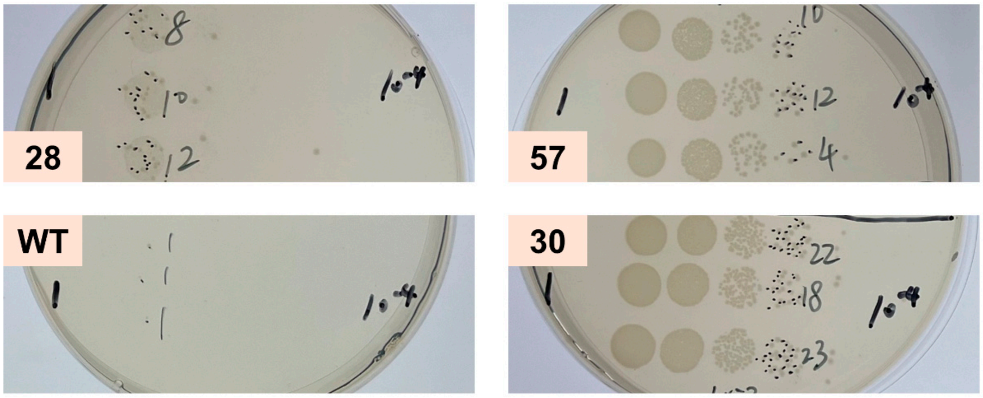

Figure S1. Representative experimental validations of off-target effects. Plasmids from the screened MAD7 library and WT-MAD7 were electroporated and, after recovery, spotted onto plates containing only the expression plasmid antibiotic.

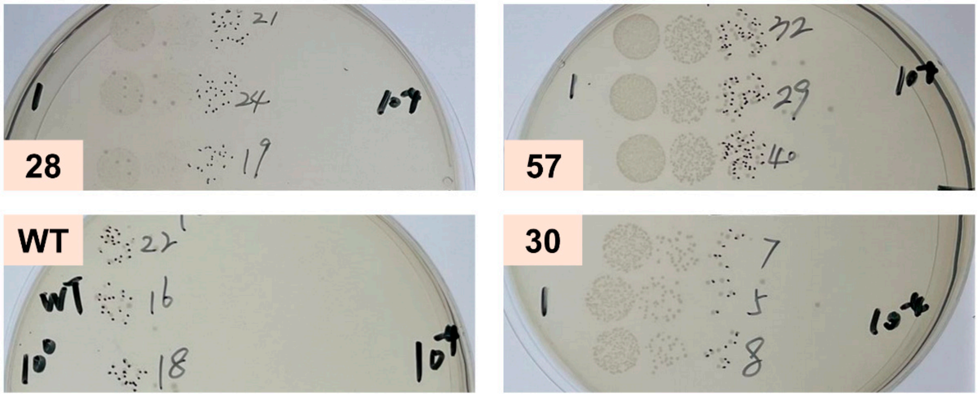

Figure S2. Representative experimental validations of on-target effects. Plasmids from the screened MAD7 library and WT-MAD7 were electroporated and, after recovery, spotted onto plates containing only the expression plasmid antibiotic and anhydrotetracycline.
